# Multi-contrast scar CINE: sparsely sampled real-time inversion-recovery bSSFP CINE combined with iterative reconstruction and motion propagation

**DOI:** 10.1186/1532-429X-17-S1-P2

**Published:** 2015-02-03

**Authors:** Michaela Schmidt, Christoph Tillmanns, Caius Fabian, Peter Speier, Michael O Zenge, Andreas Greiser, Aurelien F Stalder

**Affiliations:** 1 Siemens AG, Erlangen, Germany; 2 Diagnostikum Berlin, Berlin, Germany

## Background

A new fast and robust technique combing CINE imaging with a retrospectively adjustable delayed-enhancement (DE) contrast in a short breath-hold of 4 seconds was recently introduced [1]. As a benefit of this technique, DE images can be reconstructed as a CINE series for any TI contrast, which in turn could be beneficial in the evaluation of CMR images and allow for improved diagnostic accuracy. In this work, we performed an initial clinical evaluation of the technique.

## Methods

**Figure 1:**
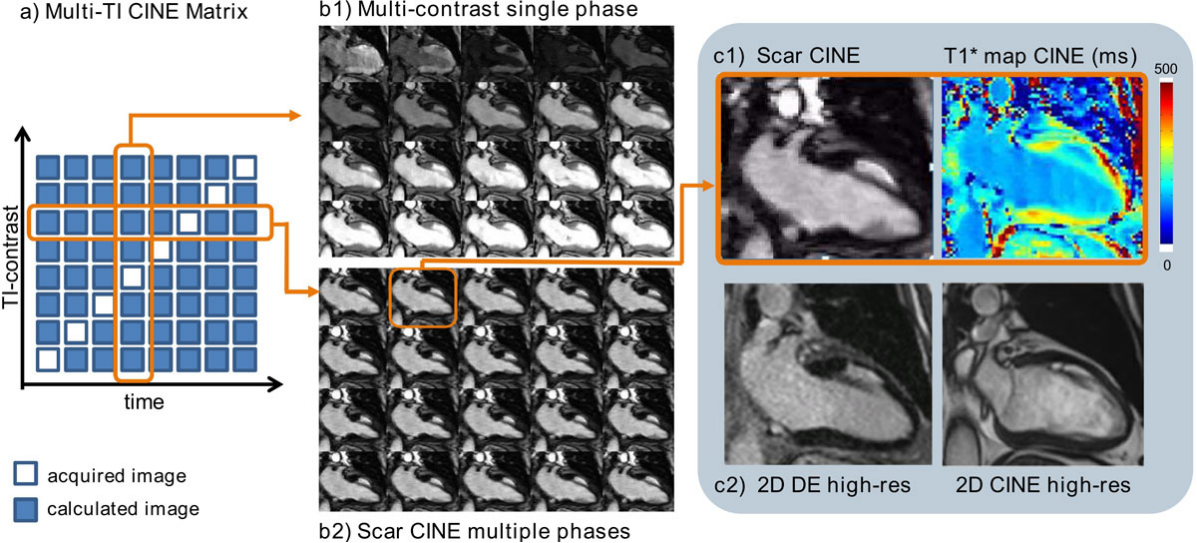
Multi-contrast scar Cine images compared to reference images in a patient with infarct in the anterior wall a) Multi-TI CINE matrix showing schematically acquired and calculated images. Calculated images were obtained by applying motion propagation for each TI contrast. b1) Example of one single cardiac phase with multiple contrasts. b2) "Scar CINE" with best myocardium-to-infarct contrast in multiple cardiac phases. c1) One diastolic phase image reconstructed from the scar CINE sequence as well as a T1* map. c2) High-resolution 2D delayed-enhancement and CINE images.

Eight patients with myocardial infarct or non-ischemic fibrosis were examined on a 3 T MR scanner (MAGNETOM Skyra, Siemens AG, Germany). Short-axis (n=5), long-axis (n=5) or both, short- and long-axis slices were acquired 8 - 15 minutes after contrast injection using the multi-contrast scar CINE prototype. This sequence consists of a 2D real-time, sparsely sampled bSSFP CINE acquired just after a non-selective inversion pulse (similar to a TI-scout) over 4 seconds during breath-hold [1]. The acquisition parameters were: TE / TR = 1.2 / 2.8 ms; approx. voxel size: 2.2 x 2.2 x 8 mm^3^; temporal resolution: 40 ms, net acceleration: 8.8. Following iterative reconstruction [2], the last cardiac cycle with almost constant contrast was used to determine the cardiac motion which was then applied to the first cardiac cycle with clinically relevant contrast changes. This motion-propagation reconstruction strategy [1] allowed for the generation of a CINE series for each of the acquired TI contrasts and a T1* map CINE (Figure 1). The results of the multi-contrast CINE were compared to high-resolution, segmented 2D CINE (approx. voxel size: 1.5 x 1.5 x 6 mm^3^) and DE images (approx. voxel size: 1.4 x 1.4 x 6 mm^3^) of the same slice. Image quality and level of diagnostic confidence to detect regional wall motion abnormality on CINE and multi-TI CINE and presence and transmurality of scar/fibrosis on DE and multi-TI CINE were graded according to a 5-point Likert-type scale by one experienced MR cardiologist and one radiologist.

## Results

**Figure 2:**
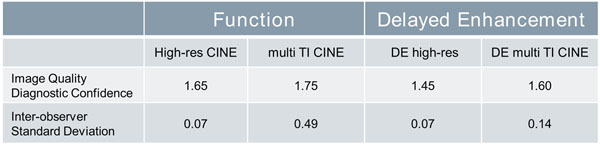
Results of image quality and level of diagnostic confidence grading according to a 5-point Likert-type scale (1=excellent, 2=good, 3=moderate, 4=poor, 5=non-diagnostic) averaged from both readers with inter-observer standard deviation.

Representative images in one patient with infarct in the anterior wall are shown in Figure 1. The image quality grading is displayed in Figure 2. The analysis of function and viability with the new scar CINE technique was diagnostic in all cases with a high image quality and diagnostic confidence score and matched the findings of the reference measurements.

## Conclusions

The proposed scar CINE which combines both the acquisition and visualization of CINE and DE data, correlates enhanced regions with altered function. The acquisition is very fast and doesn't require TI scouting. Combining function and viability with multiple TI contrasts helped to distinguish between infarct and cavum in cases with high blood pool signal or hypertrabecularization. The multi-contrast scar CINE achieved image quality scores comparable to those of the high-resolution reference scans, despite its lower resolution.

## Funding

Siemens AG.
